# Imperforate Oesophagus, Oesophageo-Tracheal Fistula

**Published:** 1914-04

**Authors:** 


Imperforate Oesophagus, Oesophageo-Tracheal Fistula. M. J. Anderodias reported a case of this nature, *Jour de Med. de Bordeaux,* October 5, 1913, although it was admitted that the fistula was not demonstrated. Nothing was done and the child died shortly after birth. Pery and Fieux reported similar cases, with demonstrated lesion. There was general agreement as to a laissez faire policy. The question of syphilis arose in all cases, as a possible explanation of the malformation.

